# The Influence of Therapeutic Radiation on the Patterns of Bone Marrow in Ovary-Intact and Ovariectomized Mice

**DOI:** 10.1371/journal.pone.0042668

**Published:** 2012-08-06

**Authors:** Susanta K. Hui, Leslie Sharkey, Louis S. Kidder, Yan Zhang, Greg Fairchild, Kayti Coghill, Cory J. Xian, Douglas Yee

**Affiliations:** 1 Department of Therapeutic Radiology, Medical School, University of Minnesota, Minneapolis, Minnesota, United States of America; 2 Department of Veterinary Clinical Sciences, College of Veterinary Medicine, University of Minnesota, St. Paul, Minnesota, United States of America; 3 Masonic Cancer Center, University of Minnesota, Minneapolis, Minnesota, United States of America; 4 Department of Medicine, Medical School, University of Minnesota, Minneapolis, Minnesota, United States of America; 5 Sansom Institute, School of Pharmacy and Medical Sciences, University of South Australia, Adelaide, South Australia, Australia; Mizoram University, India

## Abstract

**Background:**

The functional components of bone marrow (i.e., the hematopoietic and stromal populations) and the adjacent bone have traditionally been evaluated incompletely as distinct entities rather than the integrated system. We perturbed this system *in vivo* using a medically relevant radiation model in the presence or absence of ovarian function to understand integrated tissue interaction.

**Methodology/Principal Findings:**

Ovary-intact and ovariectomized mice underwent either no radiation or single fractional 16 Gy radiation to the caudal skeleton (I±R, OVX±R). Marrow fat, hematopoietic cellularity, and cancellous bone volume fraction (BV/TV %) were assessed. Ovariectomy alone did not significantly reduce marrow cellularity in non-irradiated mice (OVX−R vs. I−R, p = 0.8445) after 30 days; however it impaired the hematopoietic recovery of marrow following radiation exposure (OVX+R vs. I+R, p = 0.0092). The combination of radiation and OVX dramatically increases marrow fat compared to either factor alone (p = 0.0062). The synergistic effect was also apparent in the reduction of hematopoietic marrow cellularity (p = 0.0661); however it was absent in BV/TV% changes (p = 0.2520). The expected inverse relationship between marrow adiposity vs. hematopoietic cellularity and bone volume was observed. Interestingly compared with OVX mice, intact mice demonstrated double the reduction in hematopoietic cellularity and a tenfold greater degree of bone loss for a given unit of expansion in marrow fat.

**Conclusions/Significance:**

Ovariectomy prior to delivery of a clinically-relevant focal radiation exposure in mice, exacerbated post-radiation adipose accumulation in the marrow space but blunted bone loss and hematopoietic suppression. In the normally coupled homeostatic relationship between the bone and marrow domains, OVX appears to alter feedback mechanisms. Confirmation of this non-linear phenomenon (presumably due to differential radiosensitivity) and demonstration of the mechanism of action is needed to provide strategies to diminish the effect of radiation on exposed tissues.

## Introduction

Radiation is a common treatment modality for almost all cancers. However the impact of irradiation on normal bone and bone marrow and their interactions are not well characterized, despite the importance of understanding the complications of treatment due to the increasing number of long-term cancer survivors. Women in particular may experience accelerated bone loss from anti-cancer therapy (e.g. chemotherapy, endocrine therapy, radiation, chemo-radiation) [1], primarily due to the varying status of the ovarian hormones. Women who undergo cancer therapy experience a 10–20% increased fracture risk from treatment-induced bone loss compared to healthy patients [2], [3]. Furthermore, recovery from skeletal damage induced by radiation in women with differing ovarian function (i.e., pre- and post-menopausal) may have variable responses to anti-resorptive therapy [4]. Thus, we hypothesize that damage and subsequent recovery of the bone and bone marrow after irradiation may depend on ovarian status. This clinical question highlights the importance of fundamental biological investigations into the relationships of bone, marrow components (hemopoietic marrow measured by cellularity, stromal marrow measure by adiposity), and how they are impacted by physiological factors and the treatment of disease.

In the past, bone and hematopoietic tissues have been considered distinct entities, with bone merely providing a physical scaffold for the hematopoietic precursors of the marrow. More recent work emphasizes critical bidirectional biochemical and mechanical communications through which bone metabolism and hematopoiesis are linked [5], [6]. This suggests that a more integrated approach to the understanding of radiation-induced injury to these tissues is necessary and emphasizes the interface between basic biology and translational applications in clinical medicine. Mesenchymal stromal cells (MSC) residing in the marrow space give rise to cellular populations of the marrow microenvironment, such as osteoblasts and adipocytes. Because of their pluripotent capability, there has long been an assumption MSC phenotype directly influences bone – related parameters, i.e., increasing marrow adipogenesis is inversely proportional with bone mass [7]–[10]. MSCs additionally act as key regulators of hematopoiesis. There is evidence that MSC abnormalities contribute to the pathology of hematopoietic diseases, such as myelodysplastic syndrome [11]; that they may play a role in hematopoietic recovery after radiation injury [12]; and that they influence hematopoietic recovery after transplantation [13].

Given advancements in radiation therapy, including radiation-based conditioning regimens for bone marrow transplantation, a comprehensive evaluation of the effects on bone and marrow and their interactions will be crucial for the optimization of therapeutic protocols and the application of rational strategies for patient management. In general, marrow adiposity is shown to be negatively correlated to osteogenic potential [14]–[17]. These tissue level interactions must be clarified if more in depth investigations of molecular mechanisms are to be undertaken in terms of the relevant biology of the system in disease states. In the current study, we evaluated the effects of clinically relevant doses of radiation on patterns of bone marrow changes in murine models over time, and measured the influence of ovarian function on radiation induced changes in hematopoiesis, adipogenesis and bone mass.

## Methods

**Figure 1:**
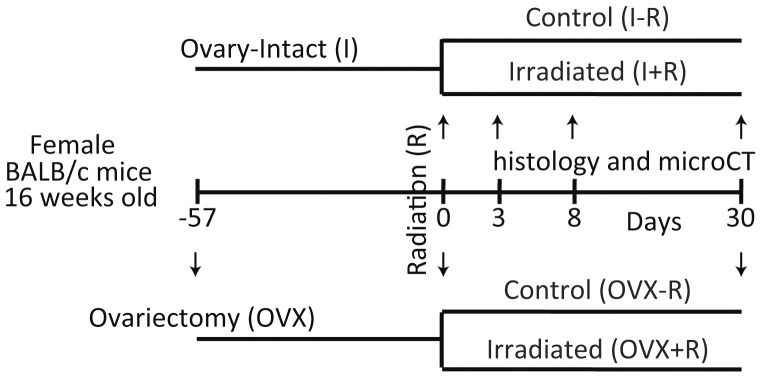
Experimental Plan Schematic. Sixteen week old BALB/c mice were ovariectomized (OVX) and maintained in the vivarium for 57 days in order to attain skeletal hemostasis. Both intact (I) and OVX mice were then irradiated with 16Gy delivered to the caudal skeleton or maintained as controls. Groups of animals were euthanized at 3, 8, and 30 days post irradiation in order to perform histological evaluations of the distal femur; microCT measurements were conducted at the 30 day time point only.

Four groups of skeletally mature BALB/c female mice (16 weeks old) were used for this study. The schematic representation of the design of this study is described by Figure 1. The first experiment characterized the response of the hematopoietic space to a clinically relevant dose of radiation in intact female mice to establish the kinetics of hematopoietic recovery in the presence of normal ovarian function. The second experiment evaluated the response of bone and hematopoietic marrow space 30 days after radiation exposure in both ovary-intact (I) and ovariectomized (OVX) mice, which were utilized to mimic the clinical management of pre-menopausal and menopausal (spontaneous or induced) cancer patients undergoing radiation therapy (R), with appropriate controls. OVX mice were ovariectomized by the vendor 57 days prior to irradiation (Jackson Laboratory; Bar Harbor, ME). The day of radiation is designated Day 0. Mice were randomly divided into the following groups: 1) “I−R” (intact no radiation, n = 5, 5 and 7 sacrificed at day 3, 8 and 30; 2) “I+R” (intact irradiated, n = 5, 5 and 9 sacrificed at day 3, 8 and 30); 3) “OVX−R” (OVX no radiation, n = 7 sacrificed at day 30); and 4) “OVX+R” (OVX irradiated, n = 8 sacrificed at day 30). This study was approved by the University of Minnesota Institutional Animal Care and Use Committee (IACUC).

### Animal Husbandry

Mice were weighed prior to irradiation and daily following irradiation for 10 days, as well as prior to placement in metabolic collection cages. Mice were housed 3–5 per cage, in a temperature- and humidity-controlled room with free access to a standard rodent chow containing 18.6% protein, 1% calcium, and 1.5 IU/g vitamin D_3_ (2018; Harlan Teklad, Madison, WI) and water. Mice were acclimated to a 12-hour light/dark cycle. There was small weight loss (0.5 to 1gm) in radiation group compared to non-irradiated group as expected. This small change in body weight however is not expected to affect the results of our study.

### Radiation Delivery

Details of radiation delivery are described by our previous publication [4]. Briefly, all mice in the I+R and OVX+R groups were anesthetized using a 0.05 mL intraperitoneal injection of a cocktail of 2.0 mL ketamine, 0.2 mL xylazine (100 mg/mL), and 1.8 mL sterile saline. All mice in the I−R and OVX−R groups were also anesthetized to control for the possible impact of anesthesia. A 16 Gy (single fraction) radiation was delivered in mice at the hind limb by a Philips RT250 orthovoltage unit. A specially designed lead shield was used to limit radiation to only the hind limbs. Proper placement of shielding was confirmed using Kodak EDR-2 film and total dose delivered was verified using micro thermo-luminescent dosimeters (micro-TLD).

### Ovariectomy verification

Wet uterine weights of mice from the I±R and OVX±R groups were measured immediately post-mortem to verify efficacy of vendor-performed ovariectomy surgeries. Ovary-intact mice had a median wet uterine weight of 0.057 g, which was significantly greater than that of 0.014 g for ovariectomized mice (Wilcoxon rank-sum test, p = 0.0003).

### MicroCT

**Figure 2:**
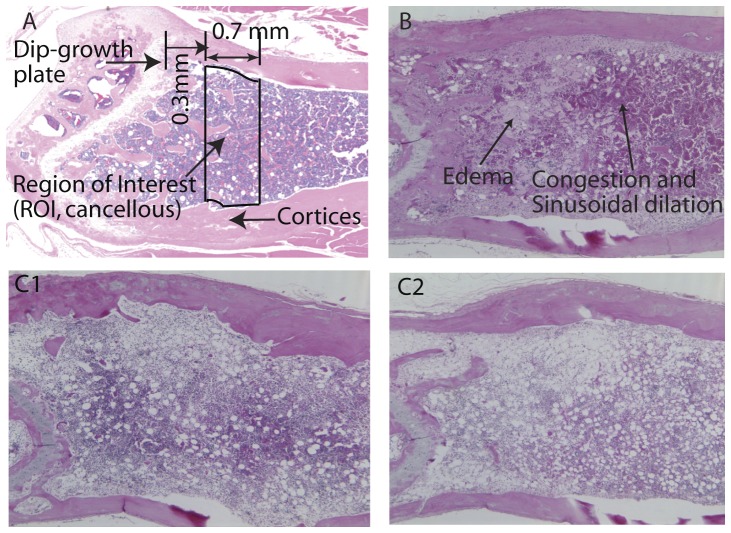
Schematic representation of bone and marrow. A. region of interest in sagittal view of a normal control mouse. B. Sagittal section of bone marrow from an intact mouse 8 days post-irradiation. Note the areas of congestion and sinusoidal dilation characterized by increased density of red blood cell within the expanded vascular spaces; also present are areas of edema characterized by increased volume of pale pink fluid in the interstitial spaces. C. Sagittal sections of bone marrow 30 days after irradiation in intact (C1) and OVX (C2) mice. Note the more intensely purple areas indicative of higher hematopoietic activity in C1 compared with C2, as well as expanded adipose in C2 compared with C1.

Bone microarchitecture of mice was analyzed with a microCT Scanner (μCT 35, Cone-Beam microCT, Scanco Medical, Switzerland). The distal femoral metaphysis was scanned within a region of 0.3–1.0 mm from the growth plate in order to avoid the primary spongiosa, with a slice thickness of 7 µm resolution (100 slices) at 70 kVp. Using the manufacturer's software, cancellous bone regions of interest (ROIs) were drawn (see Figure 2 A). The cancellous bone is composed of trabecular bone matrix and marrow. The cancellous bone volume fraction (BV/TV %; i.e. the ratio of the segmented trabecular bone volume to the total tissue volume of the region of interest) of the ROI was estimated.

### Histology

The left hind limb of each mouse was dissected. Following μCT scanning, the distal femur was collected, fixed in 10% formalin for 24 h, decalcified in Immunocal (a mild formic acid solution; Decal Corp, Tallman, NY) at 4°C until soft, processed and embedded in paraffin wax. Paraffin sections of 4 µm thick were mounted on glass slides for histological analysis. Complete sagittal sections of mouse femur were stained with hematoxylin and eosin (H&E) and were evaluated using standard procedures by a board certified veterinary clinical pathologist experienced in bone marrow evaluation (LS) [18]–[20]. Briefly, the hemopoietic cellularity (abbreviated as cellularity for the remainder of the manuscript) of the hematopoietic space was visually estimated and scored as a percentage of the total two dimensional area. The numbers of megakaryocytes were estimated as normal, mildly decreased, decreased, or markedly decreased. The ratio of myeloid to erythroid precursors (M∶E ratio) was scored as normal, mildly increased, increased, or markedly increased. If the overall cellularity was ≤5%, the numbers of hematopoietic precursors were considered too low to accurately estimate the M∶E ratio and no estimate was undertaken. Figure 2 illustrates congestion, sinusoidal dilation, and edema that were observed transiently after radiation (2B), as well as variations in hematopoietic cellularity and % adipose tissue observed on day 30 of the protocol (2C).

The maturation of hematopoietic precursors was assessed as normal or left shifted (i.e., less differentiated), indicating an abnormal number of relatively immature precursors relative to more mature forms. If the overall cellularity was ≤5%, the progression of maturation could not be accurately assessed and no evaluation was conducted. The presence of morphologic abnormalities including sinusoidal dilation, edema, congestion, inflammation, necrosis, fibrosis, vasculitis and increased hemosiderin was indicated as follows: 0 indicating abnormality was absent, 1+ mild, 2+ moderate, 3+ marked and 4+ severe changes. Endosteal lining cells were classified as normal or plump (i.e., activated).

### Marrow fat measurement

The number and percent adipocyte area (as a percentage of field) were determined from a histological section scan by a single observer using Adobe Photoshop CS5 (version 12.0.4). Reported values were then averaged for each treatment group.

### Statistical Analysis

For ovary-intact mice, two-way ANOVA with Tukey's post tests was used to evaluate the effects of radiation, time and their interaction on bone marrow hematopoietic cellularity. Megakaryocyte, myeloid: erythroid ratio, sinusoidal dilation, congestion and edema were analyzed using Kruskal-Wallis test for the comparisons across three time points in each of the I-R and I+R arms, and Wilcoxon rank-sum test for the comparisons between two time points in the same arm and between non-irradiated and irradiated arms at the same time point.

Comparisons between ovary-intact and ovariectomized mice were limited to Day 30 because ovariectomized mice had been measured for that time-point only. Wilcoxon rank-sum test was used for the comparisons between radiation treatments for the same type of mice and between different types of mice given the same radiation treatment for megakaryocyte, myeloid∶erythroid ratio, congestion and fibrosis.

To understand the interrelationships between hematopoiesis, adipogenesis and bone, two-factor multivariate analysis of variance (MANOVA) was conducted for Day 30 hematopoietic cellularity, marrow fat, and BV/TV. Factors including ovary status, radiation exposure, and their interaction were considered in the MANOVA model. Correlation between every two tissue components was evaluated by partial correlation coefficient. Following the MANOVA, two-way ANOVA was performed for individual tissue components and Tukey's post tests were used for multiple comparisons.

For Kruskal-Wallis and Wilcoxon rank-sum tests, unadjusted p values are reported and subject to adjustment by Bonferroni's method. For Tukey's post tests, adjusted p values are reported. All the analyses were conducted using SAS 9.2 (SAS Institute Inc., Cary, NC, USA).

## Results

### Time course of the hematopoietic response to radiation in intact female mice

**Figure 3:**
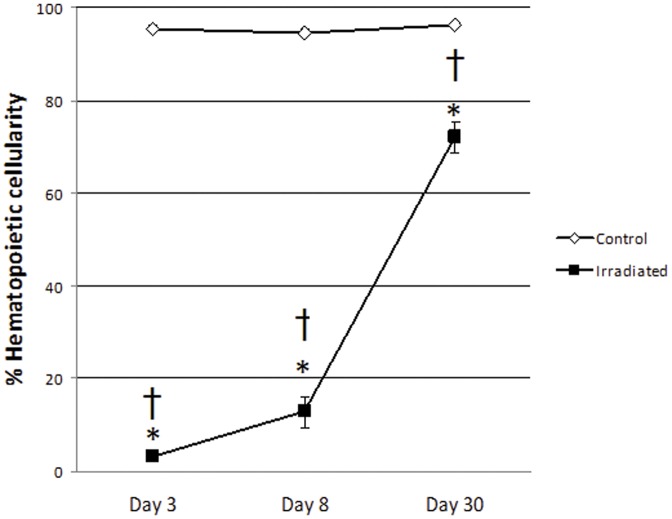
Percent of the marrow space (mean ±SEM) occupied by hematopoietic precursors in intact control (I−R) and irradiated (I+R) mice estimated by visual inspection by a board certified veterinary clinical pathologist (LS) employing standard protocols. Radiation caused marked hypocellularity of the hematopoietic components of the marrow by Day 3, with significant increases but still very low hematopoietic activity on Day 8 and marked but still incomplete recovery of hematopoietic activity with normal precursor maturation at Day 30. There were no changes over time in non-irradiated animals. Two-way ANOVA with Tukey's post-tests: * Irradiated mice had significantly lower cellularity than control mice of the same day, p<0.0001, † Cellularity of irradiated mice increased significantly from Day 3 through Day 30, p: 0.0292 – <0.0001.

In ovary-intact mice, we established that exposure to radiation resulted in marked hypocellularity of the hematopoietic components of the marrow by Day 3 (mean = 3.2%), with considerable recovery but still very low hematopoietic activity on Day 8 (mean = 12.8%, p = 0.0292 when comparing to Day 3) and marked but still incomplete recovery of hematopoietic activity with normal precursor maturation at Day 30 (mean = 72%, p<0.0001 when comparing to both Day3 and Day 8) (See Figure 3). Over the same time course, there was no change in hematopoietic cellularity in the I−R mice (mean = 95.4%, 94.6% and 96.2% for Day 3, 8 and 30, respectively) (Figure 3). As expected, megakaryocyte numbers were significantly depressed by radiation exposure on Days 3 (median scale = −3) and 8 (median scale = −3), with a trend towards ongoing suppression on Day 30 (median scale = −1) (Table 1). Although too few hematopoietic precursors were available for interpretation in the markedly hypocellular Day 3 post-irradiation marrow, the recovery phase was accompanied by significantly increased myeloid to erythroid ratios (median scale = 2 on Day 8 and = 1 on Day 30) characterized by persistent erythroid hypoplasia (Table 1). Radiation resulted in concurrent vascular abnormalities in the marrow space, including moderate to marked sinusoidal dilation and venous congestion that were mostly resolved by Day 30, and transient edema on Day 8 (Table 2). Endosteal lining cells were transiently plump in 4/5 mice on Day 3 only. Increased hemosiderin (iron pigment) was observed in 3/5 irradiated mice and fibrosis in 2/5 irradiated mice on Day 30 only. These changes were not associated with evidence of inflammation in the marrow space.

**Table 1:** Megakaryocyte number and myeloid: erythroid ratio in intact control (I−R) and irradiated (I+R) mice estimated by a board certified veterinary clinical pathologist (LS) according to standard protocols.

Treatment	Day	Megakaryocytes					Myeloid∶Erythroid ratio				
Control	3	N	N	N	N	N	N	N	N	N	N
	8	N	N	N	N	N	N	N	N	N	N
	30	N	N	N	N	N	N	N	N	N	N
Irradiated	3*	−3	−3	−3	−3	−3	NA	NA	NA	NA	NA
	8* †	−3	−3	−3	−2	−2	+2	+2	+2	+2	+2
	30* †	−1	−1	−1	−1	N	+2	+2	+1	+1	+1

Cells contain the data for individual mice (n = 5/group); N = normal, NA = too few cells to reliably estimate. Numerical scale: −4 severely decreased, −3 markedly decreased, −2 moderately decreased, −1 mildly decreased, +1 mildly increased, +2 moderately increased, +3 markedly increased, +4 severely increased, normal assigned a value of 0 for statistical calculations. Megakaryocyte numbers were significantly depressed by radiation exposure on Days 3 and 8, with a trend towards ongoing suppression on Day 30. Too few hematopoietic precursors were available for interpretation in the markedly hypocellular Day 3 post-irradiation marrow, but the recovery phase was accompanied by significantly increased myeloid to erythroid ratios characterized by persistent erythroid hypoplasia. Wilcoxon rank-sum test:

* Megakaryocytes, Irradiated significantly decreased compared with Control of the same day, Day 3, p = 0.0182; Day 8, p = 0.0236; Day 30, p = 0.0450.

† Myeloid:Erythroid ratio, Irradiation significantly increased when compared with Control, Day8, p = 0.0182; Day 30, p = 0.0236.

**Table 2:** Semi-quantitative assessment of vascular abnormalities in intact control (I−R) and irradiated (I+R) mice estimated by a board certified veterinary clinical pathologist (LS) according to standard protocols.

Treatment	Day	Sinusoidal Dilation					Congestion					Edema				
Control	3	0	0	0	0	0	0	0	0	0	0	0	0	0	0	0
	8	0	0	0	0	0	0	0	0	0	0	0	0	0	0	0
	30	0	0	0	0	0	0	0	0	0	0	0	0	0	0	0
Irradiated	3* †	+3	+3	+2	+2	+2	+2	+2	+1	+1	+3	+1	0	0	0	0
	8* † ‡	+2	+2	+2	+2	+2	+3	+2	+2	+2	+2	+2	+2	+1	+1	0
	30	+1	+1	0	0	0	+1	+1	+1	0	0	0	0	0	0	0

Numerical scale: 0 not present, +1 mild, +2 moderate, +3 marked, +4 severe. Radiation caused moderate to marked sinusoidal dilation and venous congestion on days 3 and 8 that was mostly resolved by Day 30, and transient edema on Day 8. Wilcoxon rank-sum test:

* Irradiated (marginally) significantly greater sinusoidal dilation compared with control of the same day, Day 3, p = 0.0507; Day 8, p = 0.0182.

† Irradiated significantly greater vascular congestion compared with control of the same day, Day3, p = 0.0247; Day8, p = 0.0217.

‡ Irradiated marginally significantly greater edema compared with control of the same day, Day 8, p = 0.0507.

### Hematopoietic response to radiation and OVX on day 30 post-irradiation

Radiation significantly decreased hematopoietic cellularity in intact mice (p = 0.0002), however it resulted in more severe suppression of hematopoietic cellularity in OVX mice (p<0.0001). The interaction between radiation and ovarian function was close to significance (F = 3.93, p = 0.0661) (See Table 3). Effects were similar on megakaryocytes: radiation exposure significantly decreased megakaryocyte numbers for both ovary-intact and OVX mice (p = 0.0450 and 0.0217, respectively), but the effect was exacerbated by absence of ovarian hormones (I+R vs. OVX+R, p = 0.0255) (Table 4). The myeloid to erythroid ratio was increased due to erythroid hypoplasia in irradiated mice regardless of hormonal status (p = 0.0236 and 0.0182) (Table 4). There was mild residual congestion due to irradiation in ovary-intact mice (p = 0.0495), but this was not observed in OVX mice (p = 0.1336). Irradiation-induced mild fibrosis was observed in OVX mice (p = 0.0143) but not in ovary-intact mice (p = 0.1336). Ovarian hormones did not play a significant role on the development of congestion and fibrosis with or without irradiation (p = 0.2207–1.0000 respectively) (Table 5).

**Table 3:** Comparison of group least squares means (± SEM) of hematopoietic cellularity, adiposity, and bone volume in intact (I) and ovariectomized (OVX) mice without radiation (−R) and with radiation (+R) 30 days after radiation treatment.

Treatment group	% Hematopoietic cellularity (SEM)	% Adipose (SEM)	%BV/TV (SEM)
I−R	96.2 (3.0)^a^	1.11 (0.72)^a^	5.61 (0.86)
I+R	72.0 (3.0)^a,c^	3.55 (0.72)^b^	2.63 (0.86)
OVX−R	93.0 (3.4)^b^	4.97 (0.81)^a,c^	4.53 (0.96)
OVX+R	56.0 (3.0)^b,c^	12.14 (0.72)^b,c^	3.66 (0.86)

Within columns, identical letters indicate relevant statistically significant differences between groups at p≤0.001). Radiation significantly reduced hematopoietic cellularity in intact mice, but the effect was more severe in OVX (interaction F = 4.79, p = 0.0439). OVX alone had no effect on hematopoietic activity in the absence of radiation. Radiation significantly increased marrow adipose only in OVX (interaction F = 10.14, p = 0.0062), and OVX increased marrow fat without and with radiation treatment. Radiation significantly reduced BV/TV% (p = 0.0467), but OVX had no additive effect. All statistical analyses were based on two-way ANOVA.

**Table 4:** Megakaryocyte number and myeloid: erythroid ratio in intact (I) and ovariectomized (OVX) mice receiving either 0 (−R) or 16 Gy radiation (+R) 30 days previously.

Hormone status	Radiation	Megakaryocytes					Myeloid∶Erythroid ratio				
Intact	0	0	0	0	0	0	0	0	0	0	0
	16 Gy* ‡	−1	−1	−1	−1	0	+1	+1	+1	+2	+2
OVX	0	0	0	0	0	0	0	0	0	0	0
	16 Gy* † ‡	−2	−2	−2	−2	−3	+1	+1	+1	+1	+1

Semi-quantitative estimates performed by a board certified veterinary clinical pathologist (LS) according to standard protocols. Cells contain the data for individual mice (n = 5/group). Numerical scale: −4 severely decreased, −3 markedly decreased, −2 moderately decreased, −1 mildly decreased, +1 mildly increased, +2 moderately increased, +3 markedly increased, +4 severely increased, normal assigned a value of 0 for statistical calculations. Wilcoxon rank-sum test:

* Radiation exposure significantly decreased megakaryocyte numbers compared with non-irradiated in both intact and OVX mice (p = 0.0450 and 0.0217, respectively), and

† OVX exacerbated the decrease in megakaryocytes after radiation exposure (I+R vs. OVX+R, p = 0.0255).

‡ Myeloid:erythroid ratio was increased by irradiation in both intact and OVX mice (p = 0.0236 and 0.0182, respectively).

**Table 5:** Congestion and fibrosis in intact and ovariectomized (OVX) mice receiving either 0 or 16 Gy radiation 30 days previously.

Hormone status	Radiation	Congestion					Fibrosis				
Intact	0	0	0	0	0	0	0	0	0	0	0
	16 Gy*	+1	+1	+1	0	0	+1	+1	0	0	0
OVX	0	0	0	0	0	0	0	0	0	0	0
	16 Gy†	+1	+1	0	0	0	+1	+1	+1	+1	0

Semi-quantitative estimates performed by a board certified veterinary clinical pathologist (LS) according to standard protocols. Cells contain the data for individual mice (n = 5/group). Numerical scale: −4 severely decreased, −3 markedly decreased, −2 moderately decreased, −1 mildly decreased, +1 mildly increased, +2 moderately increased, +3 markedly increased, +4 severely increased, normal assigned a value of 0 for statistical calculations. Wilcoxon rank-sum test:

* Radiation exposure significantly increased congestion compared with non-irradiated (p = 0.0495),

† Radiation significantly increased fibrosis compared with non-irradiated (p = 0.0143). Note that ovarian hormonal status did not separately affect either parameter.

### Impact of radiation and OVX on marrow fat and cancellous bone on day 30

Radiation did not significantly increase the marrow adipose content of intact mice (p = 0.1203), however radiation did significantly increase marrow adipose in OVX mice (p<0.0001). OVX significantly increased marrow adipose content in the absence of radiation (p = 0.0132) and with radiation (p<0.0001) (See Table 3). Correspondingly, the interaction between OVX and radiation significantly influenced marrow adipose level (F = 10.14, p = 0.0062). On the other hand, radiation alone significantly reduced BV/TV% (p = 0.0467), but OVX had no additive effects on BV/TV % (OVX*radiation interaction, F = 1.42, p = 0.2520). Collectively, radiation caused a disproportionate increase in marrow adiposity in OVX mice compared with intact animals, but previous ovariectomy did not influence the effect of radiation on BV/TV%, thus demonstrating a dissociation between the regulation of marrow adipose content and bone volume.

### Integration of hematopoiesis, adipogenesis, and bone volume

Overall, hematopoietic cellularity and marrow fat content were negatively correlated (partial correlation coefficient = −0.30, p = 0.2616), hematopoietic cellularity and BV/TV% were positively correlated (partial correlation coefficient = 0.12, p = 0.6587), and marrow fat and BV/TV% were negatively correlated (partial correlation coefficient = −0.13, p = 0.6328), however, none of these correlations achieved statistical significance.

**Figure 4:**
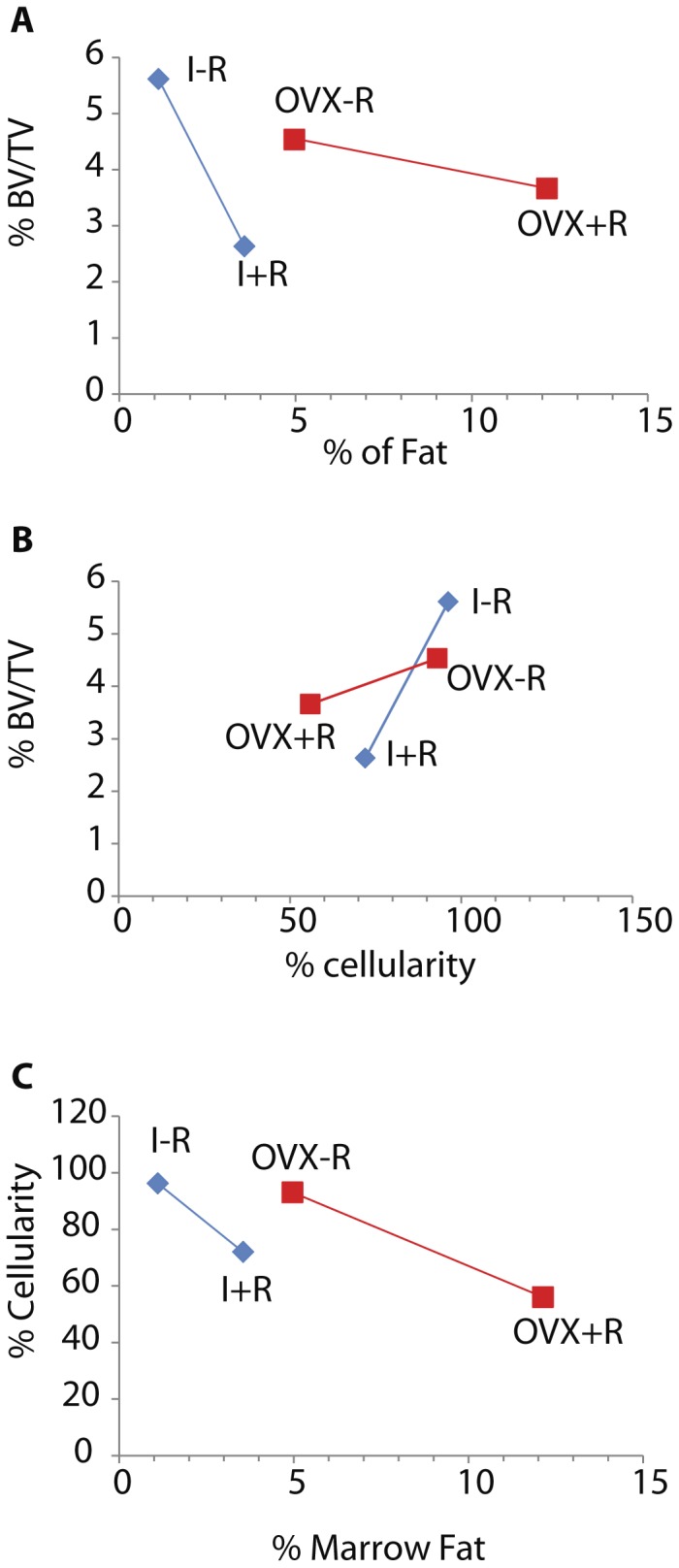
Ovariectomy influences the relationships between bone volume (BV/TV%), adipogenesis (% marrow fat), and hematopoiesis (% cellularity) at Day 30 with or without 16 Gy radiation to the hind limb in mice. Data of every two measurements are represented as least squares mean values for the group. Panel A: OVX results in a 10 fold reduction in the rate of bone volume loss per unit increase in marrow fat after radiation treatment compared with intact mice. Panel B: OVX blunts the rate of bone volume loss relative to reduced hematopoietic cellularity after radiation compared with intact mice by a factor of 6. Panel C: OVX halves the reduction in hematopoietic cellularity compared with increased marrow fat after radiation treatment compared with intact mice.

To help understand the effect of radiation in the presence or absence of ovarian function on the interrelationships between hematopoiesis, adipogenesis, and bone, we calculated radiation-induced changes in each tissue relative to the other two components for ovary-intact and OVX mice based on Figure 4. First we calculated the differences in least squares means of the three tissue components between non-irradiated and irradiated mice in the ovary intact and OVX groups, respectively. Then we calculated the following 3 ratios for each ovarian function group: difference of % BV/TV over that of % marrow fat, difference of % hematopoietic cellularity over that of % marrow fat, and difference of % BV/TV over that of % hematopoietic cellularity. Mathematically these ratios are the slopes of blue and red lines in Figure 4. They indicate the amount of change in one tissue component per unit change in another tissue component.

Specifically, intact mice demonstrate a 1.2% reduction in bone volume for every % increase in marrow adipose after radiation. OVX mice only demonstrate a 0.1% decrease in bone volume for every % increase in marrow adipose with radiation treatment. This represents a ten-fold reduction in bone volume loss per % increase in marrow fat when comparing OVX with intact mice (Figure 4, Panel A). Intact mice demonstrate a 0.1% reduction in bone volume for every 1% decrease in hematopoietic cellularity after radiation, however OVX mice show only a 0.02% reduction in bone volume for the same 1% loss of hematopoietic cellularity after radiation. OVX appears to blunt the rate of bone volume loss relative to reduced hematopoietic cellularity after radiation treatment by a factor of 6 (Figure 4, Panel B). Collectively, OVX seems to reduce the responsiveness of bone volume to changes in marrow components, with a greater change in sensitivity to adipose than hematopoiesis. Lastly, intact mice demonstrate a 9.9% reduction in hematopoietic cellularity for every 1% increase in marrow fat after radiation, while OVX results in a 5.1% reduction in hematopoietic cellularity for every 1% increase in marrow fat, approximately a half of the radiation effect for intact mice (Figure 4, Panel C).

## Discussion/Conclusions

This study was designed to gain insight into the integrated responses of bone and marrow in a laboratory rodent model of pre- and post-menopausal patients undergoing therapeutic radiation. New research strongly supports bidirectional coregulation of bone and marrow components at the micro-environmental level, attesting to the importance of evaluating these tissues as an integrated unit in order to advance our understanding of the response to injury or physiologic change [5], [21]. Our data demonstrated early and transient severe hematopoietic depletion and vascular pathology with later substantial but incomplete recovery 30 days after local irradiation in intact female mice. Furthermore, we showed that the proportionality of more chronic changes post-irradiation among the three tissue components of bone and marrow evaluated (hemopoietic, adipose, and osseous) was distinctly different depending upon the presence or absence of ovarian hormones. Multiple fold increases in the marrow fat fraction due to irradiation were not reflected in equivalent loss of either bone or hematopoietic cellularity in OVX compared with intact mice. OVX appeared to predispose the MSC population toward an adipogenic phenotype; radiation further enhanced adipogenesis in an additive fashion without proportional gross effects on the adjacent bone or hematopoietic cellularity.

As expected, irradiation in intact female mice resulted in significant and persistent decreases in hematopoietic cellularity activity of the bone marrow and increased marrow fat, although subjectively there appeared to be sufficient residual hematopoietic activity to support life in our study animals. These findings are similar to previously reported studies using male Wistar rats [22], which demonstrated early hematopoietic suppression coincident with sinusoidal dilation, and expansion of adipose in the more chronic phase (days 12–180). While specific proportional relationships were not described in detail, the authors did observe that the degree of hematopoietic recovery relative to the degree of adipogenesis varied with the radiation protocol, suggesting that the frequently observed inverse relationship of hematopoiesis and adipogenesis in the marrow does not occur in fixed ratios in all situations. Other studies in mice have specifically demonstrated the detrimental effects of radiation on the in vitro proliferation of both hematopoietic and stromal bone marrow precursor cell populations that were detectable up to six months after explant of marrow stromal cells into culture [23]. Teasing out the relative impact of radiation damage to hematopoietic and mesenchymal precursor cells is confounded by an inability to isolate subcomponents within the complex matrix of the bone marrow microenvironment [24]. In addition, repair of radiation damage may be associated with migration of HSCs as well as MSCs from non-IR treated sites [24], [25], further complicating interpretation. Due to our focus on tissue level interaction details in this manuscript, we envision series of future work in this model including mechanistic consideration for the expansion of adipose in the damaged marrow.

The current study found that OVX alone did not significantly reduce hematopoietic cellularity or bone volume while, while marrow fat content was increased. Expansion of the marrow adipose population is often associated with OVX in studies of rats and mice [26]–[28]. However, these investigators also report concurrent reductions in hematopoiesis or bone mineral density, describing a reciprocal relationship between marrow fat and hematopoiesis and bone. The degree of these changes is time dependent [27], so time and other experimental variables such as age and species or strain may explain variations in the findings. OVX may drive differentiation of the marrow stromal cells towards adipocytes at the expense of osteoblasts, resulting in diminished bone formation [28]. In contrast, other investigators describe enhanced differentiation of marrow stromal cells towards osteoblasts after OVX [29], but that bone loss is due to simultaneous but more robust increased production or activity of osteoclasts, which may actually be partially driven by the ability of osteoblasts to support osteoclastogenesis [26], [30], [31]. Preliminary histomorphometry in our laboratory indicate that OVX drives down the percent of trabecular bone surface covered by osteoblasts, though not to the same degree as irradiation with 16Gy (data not shown). In addition, we observed a significant increase in osteoclast number following OVX. Some of the effects of ovariectomy may be via canonical estrogen receptor effects on gene transcription [30], however other mechanisms may be mediated by inflammatory cells and cytokines [26], [29], [31]. The mechanistic connection with expanding adipose may be the role of fat in regulating inflammation via adipokines [26], as well as opposing effects on fat and bone of key regulatory molecules such as PPAR-γ [32].

**Figure 5:**
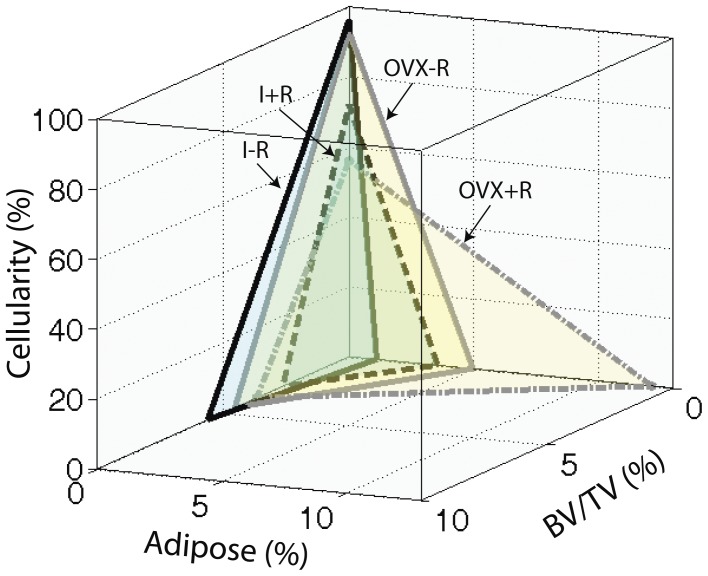
3-D illustration of interrelationships among the three tissue components of bone and marrow: hemopoietic component measured by cellularity, stromal damage component measured by marrow fat or adipose content, and osseous component measured by the cancellous bone BV/TV%. Cumulative increases in marrow fat after irradiation, in the absence of ovarian function (10 fold) was not reflected by equivalent losses of either cancellous bone or hematopoietic cellularity. The proportionality of changes in these tissue components were maintained among irradiated intact mice.

It should be noted that 30 days after irradiation was equal to 87 days post OVX in the present study (Figure 1). Since bone surfaces are generally quiescent following ovariectomy with time [33], marrow may be expected to respond to radiation to a greater degree when compared with bone, since rapidly dividing cells are more radiosensitive (demonstrated by Figures 3 and 4). Additionally, the ovary intact animals may exhibit more tightly coordinated interactions between the marrow milieu and the adjacent bone, while the lack of ovarian hormones uncouples the two resulting in disproportionate effects between these tissue envelopes as illustrated by Figure 5. New evidence demonstrates more independent regulation of bone and fat under some circumstances [34]. Differential radiosensitivity of the constituent cell populations should be investigated further by more extensive studies in bone and isolated marrow cell survivability.

Our study corroborates the inverse relationship between marrow adipose and bone described by others [32], [35], however most noticeably the stoichiometry of the association after radiation therapy is altered significantly by hormonal status. We propose complex interrelationships among the three tissue components of bone and marrow evaluated (hemopoietic, adipose, and osseous) and illustrated this in a multi-dimensional space (Figure 5). OVX and radiation had synergistic effects in promoting marrow adipogenesis and suppressing hematopoiesis. Given the reciprocal relationship between marrow fat and bone, a similar decrement in bone volume was anticipated, and this expectation was reinforced by the disproportionate hematopoietic suppression since normal osteoblastogenic signals from hematopoietic stem cells might be blunted in this population [36]. Correspondingly, osteoblasts are necessary to maintain the hematopoietic stem cell niche and support hematopoiesis, suggesting that it is reasonable to expect that the effects of radiation on hematopoiesis and bone would closely mirror one another [12], [37]. Disproportionate changes in interacting tissue components will need to be further investigated by additional studies with a larger sample size. In addition, cellular and molecular studies are necessary to help understand how ovariectomy influenced the response to radiation in this model. The ability of OVX to disrupt expected relationships between these tissues in response to radiation presents an opportunity to expand the use of this model system to understand the relationships between these closely linked tissues and has clinical implications for the treatment of cancer patients.

### Translational importance

Patients with gynecological cancer experience an accelerated and distinct pattern of trabecular bone loss from systemic chemotherapy and local pelvic radiation [1]. The murine model described by this work simulates the effects of pelvic radiation administered to pre- and post-menopausal women who have endometrial or rectal cancers. Bone marrow is considered to be a critical organ as Loren et al recently reported hematological toxicity from pelvic radiation therapy treatment [38], [39]. Different radiation modalities such as fractionated radiation therapy and total body radiation are responsible for increased commitment of non-hematopoietic mesenchymal stromal cells towards marrow adipogenesis as well as for increased bone resorption [40]–[42]. It appears that radiation (and chemotherapy) perturbs the homeostasis of bone and marrow by damaging each individual compartment (bone, hemopoietic and non-hemopoietic). Interaction of these components in presence of radiation with/without ovariectomy is unknown and will begin to unfold through stepwise increased investigations.

The experimental radiation dose is selected to be radiobiologically equivalent to the average prescribed clinical dose. A single fraction 16 Gy is radiobiologically equivalent to high dose of radiation that is given in 2Gy per fraction as hypo-fractionation. Hypo-fractionation helps repairing critical organs which is the basis of radiation therapy. Because a variety of fractionation schema is used among clinical trials, Fowler suggested using the normalized tumor dose (NTD) which is the biological effective dose (BED), normalized to a 2Gy fraction. This is discussed in details by Folwer et al extensively in their 2006 paper [43]. For late normal-tissue complications, 16 Gy (single fraction) irradiation in mice at the hind limb is approximately equivalent to 60 Gy (2 Gy/fraction) radiation dosage delivered to patients in the pelvic region. Briefly, 16 Gy is equivalent to a BED of 101.3 Gy and a NTD of 60.8 Gy, and 60 Gy (2 Gy/fraction) is equivalent to a BED of 100 Gy and a NTD of 60 Gy [using Fowler method [43] assuming α/β to be 3 Gy for late normal-tissue complications] [4]. Approximately 20% of the total murine skeleton (as measured by posthumous dry bone weight) was exposed to radiation. This is equal to the percent of total skeleton in the average irradiated pelvic region (20–30% as measured by CT) of a clinical subject. The areas of comparison between our murine model and clinical subjects also have comparable amounts of total skeletal trabecular bone volume. Therefore, as a simplified murine model, a one-time exposure of 16 Gy can be used to accurately simulate clinical irradiation of bone tissue.

Another aspect examined by the present study concerns ovarian status. We have demonstrated that the presence of ovarian hormones may modulate the severity of the radiation effect on bone and marrow. Thus, in the clinic a patient's ovarian status may be important to the ultimate preservation of bone and bone marrow integrity. As shown by Figure 5, the pre-menopausal patient may be at a higher risk for skeletal damage following clinical radiotherapy and should be more closely evaluated for irradiation effects on the skeleton. Additionally, since marrow cellularity (and presumably function) eventually recovers, hematopoietic response to treatment ought to be evaluated long-term.

### Conclusions

Therapeutic radiation affects the pattern of bone and bone marrow changes in different ways depending on the ovarian status of the recipient. Radiation results in significant damage to marrow cellularity though it recovers with time. The relationship between marrow adipogenesis and bone loss appears dependent on ovarian function, with the bone loss being greater for a given unit of expansion in marrow fat in the presence than in the absence of ovarian function. Understanding the extent and nature of changes in bone marrow, bone remodeling and related skeletal damage will be beneficial to optimizing potential interventions during or following cancer therapy.
